# 4-{2-[4-(Dimethyl­amino)­phen­yl]ethen­yl}-1-methyl­pyridinium 2,4,6-trimethyl­benzene­sulfonate monohydrate

**DOI:** 10.1107/S1600536811007690

**Published:** 2011-03-09

**Authors:** Yin Li, Jian-Xiu Zhang, Pei-Zhen Fu, Yi-Cheng Wu

**Affiliations:** a Key Laboratory of Functional Crystal and Laser Technology, Technical Institute of Physics and Chemistry, Chinese Academy of Sciences, Beijing 100190, People’s Republic of China

## Abstract

In the crystal structure of the title organic salt, C_16_H_19_N_2_^+^·C_9_H_11_O_3_S^−^·H_2_O, the cations pack head-to-tail within a sheet and are aligned in opposite directions in neighboring sheets. The benzene ring of the anion makes an angle of 76.99 (6)° with the plane of the cationic chromophore. The cations are situated in the *ab* plane, whereas the benzene rings of the anions lie in the *ac* plane.

## Related literature

For the crystal structure of solvent-free 2,4,6- trimethyl­benzene­sulfonate (DSTMS), see: Yang *et al.* (2007 ▶); Mutter *et al.* (2007 ▶). For the crystal structure of 4-*N*,*N*-dimethyl­amino-4′-*N*′-methyl­stilbazolium tosyl­ate (DAST) and DAST·H_2_O, see: Marder *et al.* (1989 ▶); Pan *et al.* (1996 ▶); Bryant *et al.* (1993 ▶). For the synthesis, see: Marder *et al.* (1994 ▶).
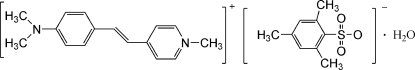


## Experimental

### 

#### Crystal data

C_16_H_19_N_2_^+^·C_9_H_11_O_3_S^−^·H_2_O
*M* *_r_* = 456.59
Triclinic, 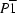

*a* = 8.1993 (17) Å
*b* = 9.669 (2) Å
*c* = 15.247 (3) Å
α = 87.806 (7)°
β = 75.805 (6)°
γ = 83.239 (5)°
*V* = 1163.7 (4) Å^3^
*Z* = 2
Mo *K*α radiation
μ = 0.17 mm^−1^
*T* = 103 K
0.50 × 0.40 × 0.20 mm

#### Data collection

Rigaku AFC10/Saturn724+ diffractometer
Absorption correction: multi-scan (*ABSCOR*; Higashi, 1995 ▶) *T*_min_ = 0.918, *T*_max_ = 0.966
11232 measured reflections
5240 independent reflections
4259 reflections with *I* > 2σ(*I*)
*R*_int_ = 0.022

#### Refinement

*R*[*F* ^2^ > 2σ(*F* ^2^)] = 0.042
*wR*(*F* ^2^) = 0.108
*S* = 1.00
5240 reflections
303 parameters
H atoms treated by a mixture of independent and constrained refinement
Δρ_max_ = 0.70 e Å^−3^
Δρ_min_ = −0.35 e Å^−3^

### 

Data collection: *CrystalClear* (Rigaku, 2008 ▶); cell refinement: *CrystalClear*; data reduction: *CrystalClear*; program(s) used to solve structure: *SHELXS97* (Sheldrick, 2008 ▶); program(s) used to refine structure: *SHELXL97* (Sheldrick, 2008 ▶); molecular graphics: *ATOMS* (Dowty, 1998 ▶); software used to prepare material for publication: *SHELXL97*.

## Supplementary Material

Crystal structure: contains datablocks I, global. DOI: 10.1107/S1600536811007690/im2263sup1.cif

Structure factors: contains datablocks I. DOI: 10.1107/S1600536811007690/im2263Isup2.hkl

Additional supplementary materials:  crystallographic information; 3D view; checkCIF report

## Figures and Tables

**Fig. 1.**
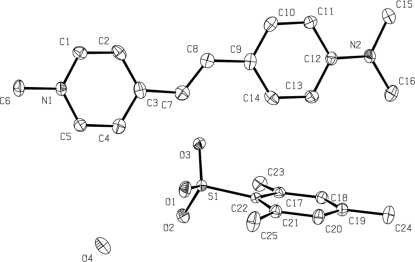
Thermal-ellipsoid (50% probability) plot showing the atomic numbering scheme.

**Fig. 2.**
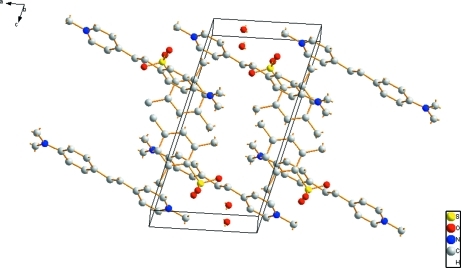
Molecular packing plot of DSTMS.H2O.

## supplementary crystallographic information

### Comment

*N,N*-Dimethyl-{4-[2-(1'-methylpyridinium-4'-yl)-vinyl]-phenyl}-amine 2,4,6- trimethylbenzenesulfonate (DSTMS) is an organic nonlinear optical crystal, which is similar to 4-*N*,*N*-dimethylamino-4'-N'- methyl-stilbazolium tosylate (DAST). Both compounds show large nonlinear optical susceptibilities (Yang *et al.*, 2007; Marder *et al.*, 1989; Pan *et al.*, 1996; Mutter *et al.*, 2007). In the presence of water, orange DSTMS hydrate with no nonlinear optical effect is easy to obtain. Fig. 1 illustrates the molecular structure of DSTMS.H_2_O together with the atomic numbering scheme. The C—H distances for the methyl groups are 0.98 Å, other H atoms are placed in idealized positions and constrained to have C—H=0.95 Å. The C—C distances for the phenyl groups range between 1.347 Å and 1.412 Å.

The unit cell of DSTMS.H_2_O contains two (C_16_H_19_N_2_)^+^ cations, two (C_9_H_11_O_3_S)^-^ anions and two water molecules, with 1 symmetry. By comparing the data of geometric parameters of solvent free DSTMS and the hydrate, there are no obvious changes of bond distances and bond angles. In both compounds, the cations group pack head to tail within a sheet and are aligned in the opposite direction in the neighboring sheets. The phenyl rings in the anion group of DSTMS.H_2_O lie at an angle of 76.99 (6)° relative to the cation chromophores, whereas the angle in the solvent free structure of DSTMS is 63.7 (2)°.

The crystal structure of DSTMS.H_2_O (Fig. 2) is analogous to DAST.H_2_O both belonging to triclinic space group *P*1 (Bryant *et al.*, 1993). DSTMS.H_2_O has two more methyl groups at the phenyl ring of the anion and the volume of unit cell therefore is larger compared with DAST.H_2_O.

### Experimental

DSTMS was synthesized by the condensation of 4-methyl-*N*-methyl pyridinium 2,4,6-trimethyl benzenesulfonate, which was prepared from 4-picoline and 2,4,6-trimethyl toluenesulfonate, and 4-*N*,*N*-dimethylamino-benzaldehyde in the presence of piperidine (Marder *et al.*, 1994). A crystal of DSTMS.H_2_O was grown by slow evaporation at room temperature from a saturated solution of DSTMS and 90% methanol/water.

### Refinement

H atoms of water were localized from Fourier maps and refined isotropically. Other H atoms were placed in calculated positions (C–H = 0.95–0.98 Å) and included in the refinement in the riding model approximation, with *U*_iso_(H) = 1.2 U_eq_(C).

### Crystal data

**Table:**

C_16_H_19_N_2_^+^·C_9_H_11_O_3_S^−^·H_2_O	*Z* = 2
*M**_r_* = 456.59	*F*(000) = 488
Triclinic, *P*1	*D*_x_ = 1.303 Mg m^−^^3^
Hall symbol: -P 1	Mo *K*α radiation, λ = 0.71073 Å
*a* = 8.1993 (17) Å	Cell parameters from 3579 reflections
*b* = 9.669 (2) Å	θ = 3.2–27.5°
*c* = 15.247 (3) Å	µ = 0.17 mm^−^^1^
α = 87.806 (7)°	*T* = 103 K
β = 75.805 (6)°	Chunk, red
γ = 83.239 (5)°	0.50 × 0.40 × 0.20 mm
*V* = 1163.7 (4) Å^3^	

### Data collection

**Table:**

Rigaku AFC10/Saturn724+ diffractometer	5240 independent reflections
Radiation source: Rotating Anode	4259 reflections with *I* > 2σ(*I*)
graphite	*R*_int_ = 0.022
Detector resolution: 28.5714 pixels mm^-1^	θ_max_ = 27.5°, θ_min_ = 3.2°
φ and ω scans	*h* = −10→10
Absorption correction: multi-scan (*ABSCOR*; Higashi, 1995)	*k* = −11→12
*T*_min_ = 0.918, *T*_max_ = 0.966	*l* = −19→19
11232 measured reflections	

### Refinement

**Table:**

Refinement on *F*^2^	Primary atom site location: structure-invariant direct methods
Least-squares matrix: full	Secondary atom site location: difference Fourier map
*R*[*F*^2^ > 2σ(*F*^2^)] = 0.042	Hydrogen site location: inferred from neighbouring sites
*wR*(*F*^2^) = 0.108	H atoms treated by a mixture of independent and constrained refinement
*S* = 1.00	*w* = 1/[σ^2^(*F*_o_^2^) + (0.0536*P*)^2^ + 0.560*P*] where *P* = (*F*_o_^2^ + 2*F*_c_^2^)/3
5240 reflections	(Δ/σ)_max_ = 0.001
303 parameters	Δρ_max_ = 0.70 e Å^−^^3^
0 restraints	Δρ_min_ = −0.35 e Å^−^^3^

### Special details

**Table:**

Geometry. All e.s.d.'s (except the e.s.d. in the dihedral angle between two l.s. planes) are estimated using the full covariance matrix. The cell e.s.d.'s are taken into account individually in the estimation of e.s.d.'s in distances, angles and torsion angles; correlations between e.s.d.'s in cell parameters are only used when they are defined by crystal symmetry. An approximate (isotropic) treatment of cell e.s.d.'s is used for estimating e.s.d.'s involving l.s. planes.
Refinement. Refinement of *F*^2^ against ALL reflections. The weighted *R*-factor *wR* and goodness of fit *S* are based on *F*^2^, conventional *R*-factors *R* are based on *F*, with *F* set to zero for negative *F*^2^. The threshold expression of *F*^2^ > σ(*F*^2^) is used only for calculating *R*-factors(gt) *etc*. and is not relevant to the choice of reflections for refinement. *R*-factors based on *F*^2^ are statistically about twice as large as those based on *F*, and *R*- factors based on ALL data will be even larger.

### Fractional atomic coordinates and isotropic or equivalent isotropic displacement parameters (Å^2^)

**Table:**

	*x*	*y*	*z*	*U*_iso_*/*U*_eq_	
S1	0.36608 (5)	0.78675 (4)	0.16984 (2)	0.01575 (11)	
O1	0.51685 (14)	0.75321 (13)	0.20319 (8)	0.0232 (3)	
O2	0.36736 (15)	0.92045 (12)	0.12167 (8)	0.0231 (3)	
O3	0.33464 (15)	0.67560 (12)	0.11702 (8)	0.0235 (3)	
N1	1.13709 (16)	0.36393 (14)	0.06835 (9)	0.0163 (3)	
N2	−0.18615 (17)	0.31865 (14)	0.36790 (9)	0.0199 (3)	
C1	1.0477 (2)	0.25780 (17)	0.06110 (11)	0.0194 (3)	
H1	1.1037	0.1764	0.0292	0.023*	
C2	0.8769 (2)	0.26638 (18)	0.09932 (11)	0.0215 (3)	
H2	0.8158	0.1908	0.0940	0.026*	
C3	0.7916 (2)	0.38704 (18)	0.14644 (11)	0.0204 (3)	
C4	0.8883 (2)	0.49457 (18)	0.15010 (11)	0.0221 (3)	
H4	0.8351	0.5788	0.1794	0.027*	
C5	1.0588 (2)	0.48092 (17)	0.11214 (11)	0.0197 (3)	
H5	1.1228	0.5549	0.1167	0.024*	
C6	1.3204 (2)	0.35286 (19)	0.02549 (11)	0.0226 (4)	
H6A	1.3378	0.3588	−0.0404	0.027*	
H6B	1.3750	0.2634	0.0421	0.027*	
H6C	1.3701	0.4290	0.0463	0.027*	
C7	0.6116 (2)	0.40354 (18)	0.19219 (11)	0.0222 (3)	
H7	0.5612	0.4933	0.2138	0.027*	
C8	0.5143 (2)	0.29984 (17)	0.20536 (11)	0.0209 (3)	
H8	0.5658	0.2119	0.1810	0.025*	
C9	0.3364 (2)	0.30837 (18)	0.25350 (11)	0.0204 (3)	
C10	0.2517 (2)	0.19128 (18)	0.25496 (11)	0.0213 (3)	
H10	0.3134	0.1078	0.2282	0.026*	
C11	0.0816 (2)	0.19291 (17)	0.29396 (10)	0.0189 (3)	
H11	0.0281	0.1110	0.2939	0.023*	
C12	−0.01440 (19)	0.31519 (16)	0.33420 (10)	0.0163 (3)	
C13	0.0716 (2)	0.43197 (16)	0.33729 (11)	0.0202 (3)	
H13	0.0117	0.5140	0.3670	0.024*	
C14	0.2427 (2)	0.42795 (17)	0.29738 (11)	0.0213 (3)	
H14	0.2983	0.5081	0.2997	0.026*	
C15	−0.2692 (2)	0.19605 (18)	0.36304 (11)	0.0221 (3)	
H15A	−0.2441	0.1659	0.3000	0.027*	
H15B	−0.3918	0.2181	0.3858	0.027*	
H15C	−0.2279	0.1212	0.4000	0.027*	
C16	−0.2850 (2)	0.44394 (19)	0.40913 (13)	0.0295 (4)	
H16A	−0.2528	0.4623	0.4650	0.035*	
H16B	−0.4056	0.4316	0.4231	0.035*	
H16C	−0.2634	0.5227	0.3671	0.035*	
C17	0.02360 (19)	0.82889 (15)	0.24753 (10)	0.0154 (3)	
C18	−0.1177 (2)	0.83123 (16)	0.32025 (11)	0.0174 (3)	
H18	−0.2270	0.8412	0.3084	0.021*	
C19	−0.1047 (2)	0.81959 (16)	0.40931 (11)	0.0183 (3)	
C20	0.0562 (2)	0.80729 (17)	0.42491 (11)	0.0199 (3)	
H20	0.0670	0.8027	0.4856	0.024*	
C21	0.2030 (2)	0.80140 (16)	0.35469 (10)	0.0178 (3)	
C22	0.18631 (19)	0.80801 (15)	0.26519 (10)	0.0143 (3)	
C23	−0.0066 (2)	0.85704 (17)	0.15439 (11)	0.0211 (3)	
H23A	−0.1263	0.8522	0.1567	0.025*	
H23B	0.0635	0.7872	0.1122	0.025*	
H23C	0.0233	0.9500	0.1338	0.025*	
C24	−0.2586 (2)	0.8222 (2)	0.48747 (11)	0.0261 (4)	
H24A	−0.3611	0.8382	0.4646	0.031*	
H24B	−0.2578	0.8972	0.5287	0.031*	
H24C	−0.2570	0.7327	0.5199	0.031*	
C25	0.3698 (2)	0.7919 (2)	0.38181 (12)	0.0287 (4)	
H25A	0.3481	0.8006	0.4476	0.034*	
H25B	0.4343	0.8671	0.3521	0.034*	
H25C	0.4350	0.7017	0.3632	0.034*	
O4	0.69606 (17)	1.00845 (14)	0.04707 (10)	0.0259 (3)	
H4A	0.611 (3)	0.977 (3)	0.0748 (18)	0.053 (8)*	
H4B	0.675 (3)	1.039 (3)	−0.0023 (18)	0.050 (7)*	

### Atomic displacement parameters (Å^2^)

**Table:**

	*U*^11^	*U*^22^	*U*^33^	*U*^12^	*U*^13^	*U*^23^
S1	0.01557 (19)	0.01519 (19)	0.01466 (18)	−0.00387 (14)	0.00108 (14)	−0.00183 (13)
O1	0.0144 (5)	0.0307 (7)	0.0228 (6)	−0.0005 (5)	−0.0015 (5)	−0.0032 (5)
O2	0.0271 (6)	0.0198 (6)	0.0196 (6)	−0.0058 (5)	0.0006 (5)	0.0033 (4)
O3	0.0215 (6)	0.0238 (6)	0.0230 (6)	−0.0079 (5)	0.0028 (5)	−0.0095 (5)
N1	0.0142 (6)	0.0215 (7)	0.0136 (6)	−0.0040 (5)	−0.0028 (5)	0.0006 (5)
N2	0.0143 (6)	0.0190 (7)	0.0240 (7)	−0.0016 (5)	0.0001 (5)	−0.0006 (5)
C1	0.0218 (8)	0.0198 (8)	0.0186 (8)	−0.0046 (6)	−0.0077 (6)	0.0004 (6)
C2	0.0218 (8)	0.0247 (8)	0.0217 (8)	−0.0100 (7)	−0.0098 (7)	0.0048 (6)
C3	0.0183 (8)	0.0276 (9)	0.0160 (7)	−0.0042 (7)	−0.0058 (6)	0.0073 (6)
C4	0.0212 (8)	0.0242 (8)	0.0198 (8)	−0.0014 (7)	−0.0031 (6)	0.0000 (6)
C5	0.0205 (8)	0.0207 (8)	0.0179 (8)	−0.0056 (6)	−0.0033 (6)	−0.0005 (6)
C6	0.0136 (7)	0.0326 (9)	0.0202 (8)	−0.0046 (7)	−0.0002 (6)	−0.0015 (7)
C7	0.0229 (8)	0.0219 (8)	0.0219 (8)	−0.0007 (7)	−0.0064 (7)	0.0010 (6)
C8	0.0200 (8)	0.0229 (8)	0.0202 (8)	−0.0011 (6)	−0.0061 (6)	0.0006 (6)
C9	0.0163 (8)	0.0277 (9)	0.0164 (7)	0.0005 (6)	−0.0041 (6)	0.0034 (6)
C10	0.0198 (8)	0.0236 (8)	0.0190 (8)	0.0026 (6)	−0.0042 (6)	−0.0025 (6)
C11	0.0203 (8)	0.0175 (8)	0.0186 (8)	−0.0010 (6)	−0.0045 (6)	−0.0016 (6)
C12	0.0159 (7)	0.0176 (7)	0.0146 (7)	−0.0010 (6)	−0.0029 (6)	0.0021 (6)
C13	0.0190 (8)	0.0157 (7)	0.0248 (8)	0.0005 (6)	−0.0046 (6)	0.0002 (6)
C14	0.0213 (8)	0.0187 (8)	0.0255 (8)	−0.0057 (6)	−0.0078 (7)	0.0048 (6)
C15	0.0199 (8)	0.0244 (8)	0.0222 (8)	−0.0057 (7)	−0.0046 (6)	0.0039 (6)
C16	0.0182 (8)	0.0279 (9)	0.0363 (10)	0.0028 (7)	0.0034 (7)	−0.0055 (8)
C17	0.0176 (7)	0.0116 (7)	0.0167 (7)	−0.0005 (6)	−0.0043 (6)	0.0004 (5)
C18	0.0151 (7)	0.0165 (7)	0.0204 (8)	−0.0010 (6)	−0.0042 (6)	−0.0005 (6)
C19	0.0170 (8)	0.0173 (8)	0.0181 (7)	−0.0018 (6)	0.0001 (6)	0.0007 (6)
C20	0.0197 (8)	0.0262 (8)	0.0132 (7)	−0.0023 (7)	−0.0027 (6)	0.0006 (6)
C21	0.0159 (7)	0.0196 (8)	0.0179 (7)	−0.0021 (6)	−0.0040 (6)	−0.0004 (6)
C22	0.0146 (7)	0.0115 (7)	0.0151 (7)	−0.0020 (5)	0.0005 (6)	−0.0014 (5)
C23	0.0228 (8)	0.0232 (8)	0.0180 (8)	−0.0004 (7)	−0.0074 (6)	0.0015 (6)
C24	0.0193 (8)	0.0344 (10)	0.0201 (8)	−0.0005 (7)	0.0025 (7)	0.0008 (7)
C25	0.0191 (8)	0.0506 (12)	0.0169 (8)	−0.0021 (8)	−0.0058 (7)	−0.0031 (8)
O4	0.0243 (7)	0.0278 (7)	0.0286 (7)	−0.0110 (5)	−0.0098 (6)	0.0085 (5)

### Geometric parameters (Å, °)

**Table:**

S1—O1	1.4468 (12)	C12—C13	1.408 (2)
S1—O3	1.4503 (11)	C13—C14	1.382 (2)
S1—O2	1.4619 (12)	C13—H13	0.9500
S1—C22	1.7969 (15)	C14—H14	0.9500
N1—C5	1.347 (2)	C15—H15A	0.9800
N1—C1	1.352 (2)	C15—H15B	0.9800
N1—C6	1.478 (2)	C15—H15C	0.9800
N2—C12	1.372 (2)	C16—H16A	0.9800
N2—C15	1.448 (2)	C16—H16B	0.9800
N2—C16	1.448 (2)	C16—H16C	0.9800
C1—C2	1.372 (2)	C17—C18	1.392 (2)
C1—H1	0.9500	C17—C22	1.415 (2)
C2—C3	1.411 (2)	C17—C23	1.510 (2)
C2—H2	0.9500	C18—C19	1.387 (2)
C3—C4	1.390 (2)	C18—H18	0.9500
C3—C7	1.463 (2)	C19—C20	1.388 (2)
C4—C5	1.369 (2)	C19—C24	1.507 (2)
C4—H4	0.9500	C20—C21	1.398 (2)
C5—H5	0.9500	C20—H20	0.9500
C6—H6A	0.9800	C21—C22	1.403 (2)
C6—H6B	0.9800	C21—C25	1.515 (2)
C6—H6C	0.9800	C23—H23A	0.9800
C7—C8	1.333 (2)	C23—H23B	0.9800
C7—H7	0.9500	C23—H23C	0.9800
C8—C9	1.457 (2)	C24—H24A	0.9800
C8—H8	0.9500	C24—H24B	0.9800
C9—C10	1.393 (2)	C24—H24C	0.9800
C9—C14	1.405 (2)	C25—H25A	0.9800
C10—C11	1.374 (2)	C25—H25B	0.9800
C10—H10	0.9500	C25—H25C	0.9800
C11—C12	1.412 (2)	O4—H4A	0.81 (3)
C11—H11	0.9500	O4—H4B	0.85 (3)
			
O1—S1—O3	112.80 (7)	C12—C13—H13	119.8
O1—S1—O2	111.61 (7)	C13—C14—C9	121.66 (15)
O3—S1—O2	112.36 (7)	C13—C14—H14	119.2
O1—S1—C22	108.39 (7)	C9—C14—H14	119.2
O3—S1—C22	105.54 (7)	N2—C15—H15A	109.5
O2—S1—C22	105.59 (7)	N2—C15—H15B	109.5
C5—N1—C1	120.26 (14)	H15A—C15—H15B	109.5
C5—N1—C6	120.03 (13)	N2—C15—H15C	109.5
C1—N1—C6	119.69 (13)	H15A—C15—H15C	109.5
C12—N2—C15	119.93 (13)	H15B—C15—H15C	109.5
C12—N2—C16	120.42 (14)	N2—C16—H16A	109.5
C15—N2—C16	119.65 (14)	N2—C16—H16B	109.5
N1—C1—C2	120.76 (15)	H16A—C16—H16B	109.5
N1—C1—H1	119.6	N2—C16—H16C	109.5
C2—C1—H1	119.6	H16A—C16—H16C	109.5
C1—C2—C3	120.28 (15)	H16B—C16—H16C	109.5
C1—C2—H2	119.9	C18—C17—C22	118.60 (14)
C3—C2—H2	119.9	C18—C17—C23	117.57 (14)
C4—C3—C2	116.78 (15)	C22—C17—C23	123.74 (14)
C4—C3—C7	119.04 (15)	C19—C18—C17	122.41 (15)
C2—C3—C7	124.16 (15)	C19—C18—H18	118.8
C5—C4—C3	121.03 (16)	C17—C18—H18	118.8
C5—C4—H4	119.5	C18—C19—C20	117.74 (14)
C3—C4—H4	119.5	C18—C19—C24	121.92 (15)
N1—C5—C4	120.85 (15)	C20—C19—C24	120.33 (15)
N1—C5—H5	119.6	C19—C20—C21	122.50 (15)
C4—C5—H5	119.6	C19—C20—H20	118.7
N1—C6—H6A	109.5	C21—C20—H20	118.7
N1—C6—H6B	109.5	C20—C21—C22	118.52 (14)
H6A—C6—H6B	109.5	C20—C21—C25	116.73 (14)
N1—C6—H6C	109.5	C22—C21—C25	124.74 (14)
H6A—C6—H6C	109.5	C21—C22—C17	120.04 (14)
H6B—C6—H6C	109.5	C21—C22—S1	122.28 (12)
C8—C7—C3	123.74 (16)	C17—C22—S1	117.66 (11)
C8—C7—H7	118.1	C17—C23—H23A	109.5
C3—C7—H7	118.1	C17—C23—H23B	109.5
C7—C8—C9	126.30 (16)	H23A—C23—H23B	109.5
C7—C8—H8	116.9	C17—C23—H23C	109.5
C9—C8—H8	116.8	H23A—C23—H23C	109.5
C10—C9—C14	117.34 (15)	H23B—C23—H23C	109.5
C10—C9—C8	118.18 (15)	C19—C24—H24A	109.5
C14—C9—C8	124.46 (16)	C19—C24—H24B	109.5
C11—C10—C9	121.99 (15)	H24A—C24—H24B	109.5
C11—C10—H10	119.0	C19—C24—H24C	109.5
C9—C10—H10	119.0	H24A—C24—H24C	109.5
C10—C11—C12	120.65 (15)	H24B—C24—H24C	109.5
C10—C11—H11	119.7	C21—C25—H25A	109.5
C12—C11—H11	119.7	C21—C25—H25B	109.5
N2—C12—C13	121.89 (14)	H25A—C25—H25B	109.5
N2—C12—C11	120.30 (14)	C21—C25—H25C	109.5
C13—C12—C11	117.81 (14)	H25A—C25—H25C	109.5
C14—C13—C12	120.40 (15)	H25B—C25—H25C	109.5
C14—C13—H13	119.8	H4A—O4—H4B	105 (2)
			
C5—N1—C1—C2	−1.1 (2)	C12—C13—C14—C9	−0.6 (2)
C6—N1—C1—C2	−179.16 (14)	C10—C9—C14—C13	−2.6 (2)
N1—C1—C2—C3	0.4 (2)	C8—C9—C14—C13	176.07 (15)
C1—C2—C3—C4	1.1 (2)	C22—C17—C18—C19	2.7 (2)
C1—C2—C3—C7	−177.60 (15)	C23—C17—C18—C19	−173.92 (14)
C2—C3—C4—C5	−2.1 (2)	C17—C18—C19—C20	1.0 (2)
C7—C3—C4—C5	176.71 (15)	C17—C18—C19—C24	−179.96 (15)
C1—N1—C5—C4	0.1 (2)	C18—C19—C20—C21	−2.3 (2)
C6—N1—C5—C4	178.19 (15)	C24—C19—C20—C21	178.59 (15)
C3—C4—C5—N1	1.5 (3)	C19—C20—C21—C22	−0.1 (2)
C4—C3—C7—C8	−168.78 (16)	C19—C20—C21—C25	178.41 (16)
C2—C3—C7—C8	9.9 (3)	C20—C21—C22—C17	3.9 (2)
C3—C7—C8—C9	177.35 (15)	C25—C21—C22—C17	−174.51 (15)
C7—C8—C9—C10	175.50 (16)	C20—C21—C22—S1	−174.67 (12)
C7—C8—C9—C14	−3.2 (3)	C25—C21—C22—S1	7.0 (2)
C14—C9—C10—C11	2.8 (2)	C18—C17—C22—C21	−5.1 (2)
C8—C9—C10—C11	−175.95 (15)	C23—C17—C22—C21	171.28 (14)
C9—C10—C11—C12	0.2 (2)	C18—C17—C22—S1	173.46 (11)
C15—N2—C12—C13	179.87 (15)	C23—C17—C22—S1	−10.1 (2)
C16—N2—C12—C13	−0.7 (2)	O1—S1—C22—C21	4.19 (15)
C15—N2—C12—C11	0.6 (2)	O3—S1—C22—C21	125.28 (13)
C16—N2—C12—C11	−179.95 (15)	O2—S1—C22—C21	−115.53 (13)
C10—C11—C12—N2	175.85 (14)	O1—S1—C22—C17	−174.38 (11)
C10—C11—C12—C13	−3.4 (2)	O3—S1—C22—C17	−53.28 (13)
N2—C12—C13—C14	−175.66 (15)	O2—S1—C22—C17	65.90 (13)
C11—C12—C13—C14	3.6 (2)		
